# Multicellular Cancer-Stroma Spheres (CSS) for In Vitro Assessment of CAR-T Cell-Associated Toxicity

**DOI:** 10.3390/cells13221892

**Published:** 2024-11-16

**Authors:** Aigul R. Rakhmatullina, Mariya A. Zolotykh, Yuliya V. Filina, Aigul Kh. Valiullina, Ekaterina A. Zmievskaya, Dina U. Gafurbaeva, Aisylu R. Sagdeeva, Emil R. Bulatov, Albert A. Rizvanov, Regina R. Miftakhova

**Affiliations:** 1 Institute of Fundamental Medicine and Biology, Kazan Federal University, 420008 Kazan, Russia; 2 Division of Medical and Biological Sciences, Academy of Sciences of the Republic of Tatarstan, 420111 Kazan, Russia

**Keywords:** CAR-T therapy, cytokine release syndrome, immune effector cell-associated neurotoxicity syndrome, cancer-stroma spheres, mesenchymal stem cell, IL-8, MCP-1, IP-10

## Abstract

CAR-T therapy has revolutionized the field of oncology, offering a promising treatment option for cancer patients. However, the significant morbidity associated with therapy-related toxicity presents a major challenge to its widespread use. Despite extensive research into the underlying mechanisms of CAR-T therapy-related toxicity, there are still many unknowns. Furthermore, the lack of adequate in vitro models for assessing immunotoxicity and neurotoxicity further complicates the development of safer cellular therapies. Previously in our laboratory, we developed cancer-stroma spheres (CSS) composed of prostate adenocarcinoma PC3 cells and mesenchymal stem cells (MSC). Herein we present evidence that multicellular CSS could serve as a valuable in vitro model for toxicity studies related to CAR-T therapy. CSS containing CD19-overexpressing PC3M cells exhibited increased secretion of CAR-T cell toxicity-associated IL-8, MCP-1, and IP-10 in the presence of anti-CD19 CAR-T cells, compared to spheres derived from single cell types.

## 1. Introduction

Chimeric antigen receptor (CAR)-T cell therapy is one of the most actively developing areas of immuno-oncology. The high efficiency of CAR-T therapy has been shown for the treatment of B-cell acute lymphoblastic leukemia (B-cell ALL), lymphoma, and multiple myeloma, which resulted in FDA approval of six cellular therapies [1,2]. However, the effectiveness of this therapy is countered by a significant risk of systemic and life-threatening side effects. Common CAR-T-mediated toxicities include cytokine release syndrome (CRS) and neurotoxicity, referred to as immune effector cell-associated neurotoxicity syndrome (ICANS) [1,3].

Incidences of CRS and ICANS reported in pivotal clinical trials range from 42% to 95% and 4% to 64%, respectively (Table 1). Severe-grade toxicity can provoke the development of multiple organ dysfunction syndromes that can lead to death. The review of data on more than 1000 patients with B-cell lymphoma indicated that 7–10% of deaths occurring within 30 days after CAR-T infusion were unrelated to disease relapse [4].

### 1.1. CRS

CRS is associated with an inflammatory response induced by CAR-T cells. Clinical manifestation of CRS includes fever, organ dysfunction, and hypotension [5]. Severe CRS (grade 3 and worse) occurred in 24–46% of patients with B-cell ALL [6] and in 3–23% of patients with B-cell lymphoma [7,8] in phase 2 CD19-CART clinical trials (Table 1).

The serum levels of the main inflammatory cytokines including interleukin-6 (IL-6), tumor necrosis factor α (TNFα), interferon gamma (IFN-γ), monocyte chemoattractant protein 1 (MCP-1), granulocyte-macrophage colony-stimulating factor (GM-CSF), interleukin-1 (IL-1), and interleukin-8 (IL-8) have been found elevated in patients with hematological malignancies after CAR-T infusion [9,10]. Furthermore, cytokine levels correlate with the severity of CRS [11]. The study of Teachey et al. was focused on predicting severe CRS development upon CD19-CAR-T stimulation in patients with relapsed/refractory ALL. The highest grade of CRS in a cohort of 51 patients was most accurately predicted by the combination of IFNγ, soluble gp130, and soluble receptor antagonist IL1 (sIL1RA) with a sensitivity of 86% and a specificity of 89% in the logistic regression model [12].

**Table 1:** FDA-approved CAR-T therapies and the frequency of side effects.

CAR-T	Target	Trial	Condition	Number of Patients	Cytokine Release Syndrome		Neurotoxicity (ICANS)		References
					Any Grade,%	≥3 Grade, %	Any Grade,%	≥3 Grade, %	
KIMRYAH	CD19	JULIET	Adult r/r DLBCL	111	58	22	21	12	[8]
		ELIANA	Pediatric r/r B-ALL	79	77	46	40	13	[6]
		ELARA	r/r FL	97	49	0	37(4)	3 (1)	[13]
YESCARTA	CD19	ZUMA-1	r/r LBCL	101	93	13	64	28	[7]
TECARTUS	CD19	ZUMA-2	r/r MCL	68	91	15	63	31	[14]
		ZUMA-3	r/r B-ALL	55	89	24	60	25	[15]
BREYANZI	CD19	TRANSCEND NHL 001	r/r LBCL	269	42	2	30	10	[16]
		TRANSCEND FL	r/r FL	101	58	1	15	2	[17]
		TRANSCEND CLL 004	r/r CLL	96	85	9	45	19	[18]
ABECMA	BCMA	KarMMa-3	r/r MM	225	88	5	15	3	[19]
CARVYKTI	BCMA	CARTITUDE-1	r/r MM	97	95	5	22	12	[20]

More in-depth studies show that cytokine dynamics depend on the timing and intensity of inflammation. For example, research by Wei and colleagues categorized post-CAR-T inflammation into three stages: local inflammation, systemic inflammation, and inflammation associated with organ dysfunction [21]. Local inflammation has been linked to elevated levels of TNF-a, IFNg, CD40L, and GM-CSF, which are related to the interaction of CAR-T cells with target cells. First-stage cytokines, for example GM-CSF and CD40L, further activate macrophages [22,23]. Macrophage-derived IL-6 and partially TNFa affect endothelial cells, inducing vascular permeability which results in the initiation of the second stage of CRS. Systemic inflammation has been associated with increased levels of IL-1 and IL-6. And organ dysfunction stage is characterized by a rise in serum amyloid P (SAP), C-reactive protein (CRP), further increase in serum IL1 and IL6.

### 1.2. ICANS

ICANS often occurs after CRS and rarely appear independent from CRS, suggesting that the driver factors of these two syndromes overlap [24]. In a study by Santomasso et al., the first neurological symptoms were detected at a median of 5 days post-CAR-T infusion, with the median time to the first severe neurotoxicity being 9 days in patients with B-ALL [3].

ICANS was characterized by delirium, decreased level of consciousness, and language impairment. Seizures, motor weakness, cerebral oedema, and coma have been correlated with severe cases of ICANS [25]. Most studies report increased serum levels of IL-6, IFN-y, TNF-a, IL-2, IL-15, IL-4, and HGF [25,26]. In a study involving 133 adults, of whom 40% developed neurotoxicity after treatment with anti-CD19 CAR-T cells, a correlation was found between toxicity grades and endothelial activation: severe ICANS was accompanied by increased levels of angiopoietin and von Willebrand factor. Moreover, the increase in concentrations of systemic cytokines IL-6, IFNγ, and TNFα has been found in spinal fluid, which was found to be related to disruption of the blood–brain barrier. In turn, IFNγ and TNFα induced secretion of systemic and endothelium-activating cytokines by pericytes [25]. The emerging high levels of IL-6, IL-8, MCP1, and IP-10 have been observed in the spinal fluid of patients with B-ALL, which were disproportionate to white blood cell or CAR-T cell counts. The authors concluded on the evidence of the production of cytokines by central nervous system cells [27].

### 1.3. Test Systems for CAR-T Toxicity Screening

To date, there is a lack of in vitro models capable of assessing the initial toxicity of CAR-T therapy. Most preclinical studies have used tumor cell cultures as target cells for CAR-T and performed cytokine analysis in cell culture supernatants [28]. Considering that toxicity is a result of interactions between CAR-T cells and endogenous cells of the body, in particular immune cells, multicellular systems have shown to be more successful models for toxicity assessment.

For example, co-culture of tumor cell lines with autologous monocytes resulted in a significant elevation of IL-6 level in two out of the three tested CAR-T therapeutics compared to tumor cell culture alone [29]. At the same time, IL-2 and IL-1Ra levels were not affected by monocyte presence in a culture.

Another study compared a combination of monocytes and two types of CAR-T cells: conventional and those obtained by a rapid protocol [30]. Rapid CAR-T cells then cultured with monocytes show a significant increase in IL-6, IFN-γ, TNF-α, GM-CSF, IL-2, and IL-10 levels, as compared to conventional CAR-T—monocyte culture. CRS prediction results were confirmed in in vivo experiments where rapid CAR-T cells, but not conventional ones, induced hypothermia, weight loss, and elevation of murine MCP-1, IL-6, and G-CSF. Elevation of human IFN-γ, TNF-α, IL-2, and IL-10 cytokines in mice serum indicated the direct role of CAR-T cells in their hyperproduction.

Among in vivo models, humanized mouse models are the most suitable for immunological studies [31,32]. In a study involving 120 NSG mice, the authors illustrate how the levels of key humanized cytokines such as IL-2, IL-4, IL-6, IL-10, IFN-γ, and TNF can vary throughout the therapeutic process, providing valuable insights into the dynamics of CAR-T cell activity [32]. Additionally, humanized mice with patient-derived xenografts from acute lymphoblastic leukemia (ALL) were used to highlight the critical roles of GM-CSF, IL-18, MIP-1α, and IP-10 in the CAR-T response [33]. However, the use of animal models can be costly and requires specific maintenance conditions. Tumor spheres are three-dimensional cellular in vitro models that provide better reproducibility of results from in vivo studies. Tumor spheres and solid tumors exhibit comparable histological patterns when sectioned, as well as internal gradients of signaling factors and nutrients [34]. Previously, we designed a complex cancer-stroma sphere (CSS) model using prostate cancer cells and MSCs as the stromal component of the tumor [35]. The conducted study aimed to test whether the CSS model has benefits compared with single-cell type sphere models in assessing CAR-T treatment efficacy and toxicity and whether CSS can be applied to elucidate the role of MSC in CAR-T-associated toxicity.

## 2. Materials and Methods

### 2.1. Cell Cultures

Immortalized MSC (hereafter MSC-GFP or MSC) were previously produced in our laboratory through the overexpression of the hTERT gene and the knockdown of TP53 [36]. Additionally, prostate cancer cell line 3 overexpressing CD19 antigen (hereafter PC3M-CD19-Katushka or PC3M) was early generated in our laboratory by lentiviral transduction [37]. CAR-T cells (hereafter anti-CD19 CAR-T cells) were generated using lentiviral transduction of second-generation CAR (FMC63-28Z) according to previously reported protocol [37]. The generation of CAR-T cells was conducted in accordance with the Declaration of Helsinki and approved by the local ethics committees of the Kazan Federal University, Kazan, Russia (Approval Code: No. 28, Date 25 March 2021).

All cell types (PC3M, MSC, anti-CD19 CAR-T) were cultured in full RPMI-1640 medium (PanEco, Moscow, Russia) in the presence of 10% fetal bovine serum (FBS) (Hyclone, Logan, UT, USA), 2 mM L-glutamine, 50 U/mL penicillin, and 50 μg/mL streptomycin (PanEco, Moscow, Russia) at 37 °C, and 5% CO_2_.

### 2.2. Sphere-Formation Assay

DMEM/F-12 media (PanEco, Moscow, Russia) were mixed with B-27 neuronal culture supplement (2×) (PanEco, Moscow, Russia), 40 ng/mL EGF (Sci-store, Moscow, Russia), and 40 ng/mL FGF2 (Sci-store, Moscow, Russia) to create sphere formation media. A pre-optimized concentration of tumor cells (1000 cells/mL) was combined with MSC (5000 cells/mL) to create CSS. Spheres were grown in sphere formation media on low attachment cell culture dishes. The detection and imaging of spheres were performed using an Axio Observer.Z1 fluorescence microscope (Carl Zeiss, Oberkochen, Germany), equipped with FITC and Cy5 filter sets on the 7th day of culture.

### 2.3. Sphere Treatment with Anti-CD19 CAR-T-Cells

Sphere formation was assessed in 35 mm low attachment cell culture dishes in a sphere formation media according to the protocol described in Section 2.2. After sphere detection on day 7 of culture, anti-CD19 CAR-T cells were added to all sphere types (PC3M, MSC, CSS) at a density of 10^6^ cells per dish. Spheres were visualized and counted 6 and 24 h post anti-CD19 CAR-T-cells addition on an Axio Observer.Z1 fluorescence microscope (Carl Zeiss, Oberkochen, Germany). After 6 h of incubation with anti-CD19 CAR-T cells, the culture medium was collected for multiplex immunoassay analysis.

### 2.4. Flow Cytometry Analysis of Cell Death

Control and anti-CD19 CAR-T-treated MSC, PC3M spheres, and CSSs were harvested by centrifugation at 400× *g* for 5 min. Single cells were prepared by dissociating pellets after preincubation with trypsin-EDTA 0.25% (PanEco, Moscow, Russia) for 10 min at 37 °C. Following trypsin neutralization, the cells were resuspended in cold Annexin V binding buffer (#422201, Biolegend, San Diego, CA, USA) and stained with Annexin V (#640947, Biolegend, San Diego, CA, USA) and 7-AAD (#420404, Biolegend, San Diego, CA, USA) according to the manufacturer’s instructions. Apoptotic and necrotic populations were analyzed using BD FACSAria™ III flow cytometer (BD Biosciences, Franklin Lakes, NJ, USA).

### 2.5. Analysis of Cytokine Levels in Conditioned Media

The quantitative analysis of cytokine and chemokine levels in sphere-conditioned media after 6 h of anti-CD19 CAR-T-cells addition was conducted using bead-based multiplex technology. The Human Cytokine/Chemokine Magnetic Bead Panel (#HCYTMAG-60K-PX41, Merck Millipore, Burlington, MA, USA) was employed to detect 41 analytes in 25 microliters of media. Prior to analysis, the conditioned media were thawed on ice, and all subsequent steps were performed following the manufacturer’s recommendations. The MAGPIX System with xPONENT 4.2 (Luminex, Austin, TX, USA) was utilized to register median fluorescence intensities, adhering to a 50-bead count requirement. Data analysis was performed using Bio-Plex Manager 6.1 Software (Bio-Rad, Hercules, CA, USA).

### 2.6. Statistical Analysis

Statistical analyses were performed using GraphPad Prism 8.0 Software (Graphpad Software, LLC Company Profile, Boston, MA, USA). The comparison of means between different groups was performed using ANOVA with appropriate post-hoc tests. Data are representative of at least three independent experiments and shown as mean ± SEM otherwise specified; *p*-values are indicated as *—≤0.05, **—≤0.01, ***—≤0.005, ****—≤0.0001.

## 3. Results

### 3.1. Anti-CD19 CAR-T Cells Demonstrate Cytotoxic Effect on PC3M and CSS Spheres

**Figure 1:**
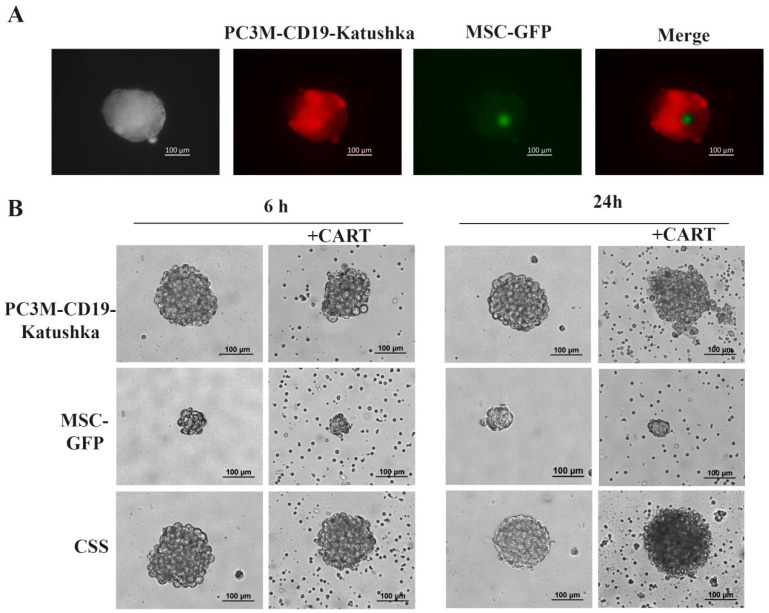
Microphotography of spheres formed by tumor, stroma cells, and their co-culture: (**A**) representative fluorescent microphotographs of CSS; (**B**) representative microphotographs of PC3M, MSC spheres, and CSSs treated by anti-CD19 CAR-T cells after 6 and 24 h of incubation.

The presence of tumor cells and MSCs in the CSS was confirmed via fluorescent microscopy through the detection of fluorescent proteins pKatushka2S and GFP, respectively. MSCs were detected in the center of CSS, while tumor cells were located at the periphery of the spheres (Figure 1A). Both PC3M spheres and CSS exhibited sizes greater than 100 μm (185.01 ± 36.04 μm and 177.55 ± 24.20 μm, respectively), whereas MSCs were smaller, measuring less than 100 μm (88.87 ± 11.49 μm). Additionally, all types of spheres displayed clear boundaries and exhibited a compact structure (Figure 1B).

The morphology of the spheres remained unchanged after 6 h of incubation with anti-CD19 CAR-T cells. However, after 24 h, both PC3M spheres and CSS began to disintegrate into small cell aggregates, with the cell cytoplasm becoming poorly visualized, indicating cell death in the outer layer of the sphere (Figure 1B).

**Figure 2:**
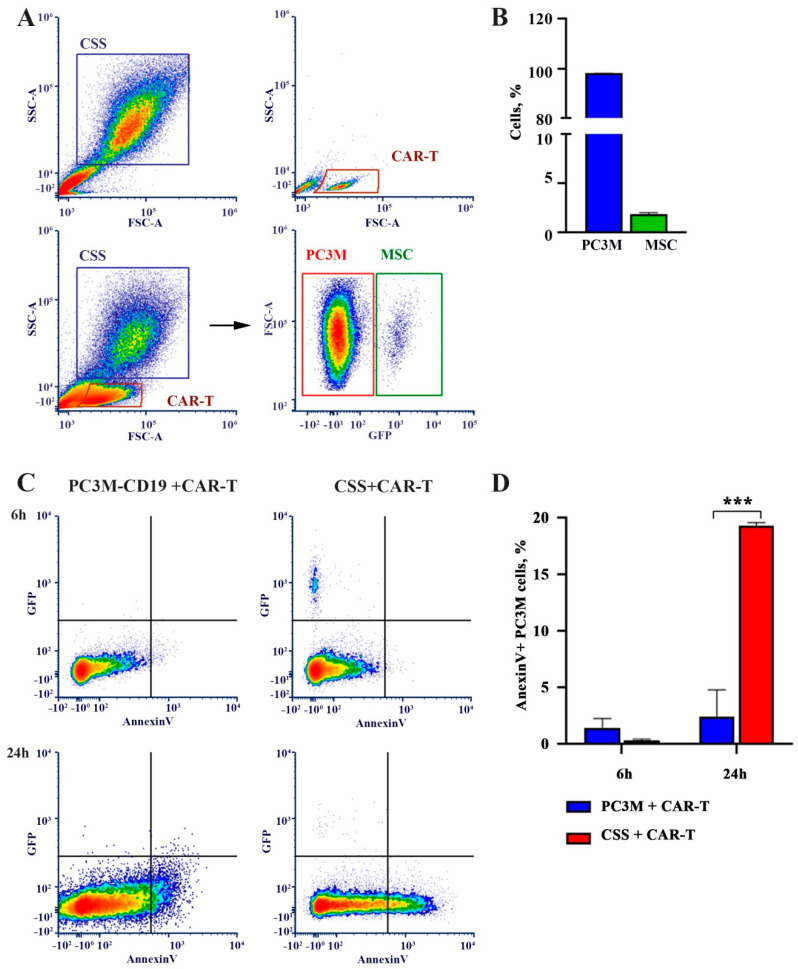
Analysis of cytotoxic effect of anti-CD19 CAR-T: (**A**) gating strategy to discriminate PC3M, MSC, and anti-CD19 CAR-T cells represented by flow cytometry plots; (**B**) proportion of PC3M and MSC in CSS; (**C**) representative flow cytometry plots with apoptotic cell gating strategy; (**D**) percent of Annexin V+ cells in PC3M spheres and CSS ( *p*-value is indicated as *** – ≤0.005). The data represents mean ± standard error of three independent experiments.

We also assessed the effect of anti-CD19 CAR-T on tumor cell viability using an Annexin V/7-AAD staining, which was performed on cells after 6 and 24 h of anti-CD19 CAR-T incubation. The populations of PC3M, MSC, and anti-CD19 CAR-T cells were identified based on their size and granularity as shown in the forward and side light scattering plot (Figure 2A). MSC in CSS was identified using GFP fluorescence (Figure 2A). In the CSS, 97.97 ± 0.17% of the cells were presented by PC3M cells, while 1.88 ± 0.15% were identified as MSCs (Figure 2B). No significant difference in cell viability was detected after 6 h of anti-CD19 CAR-T treatment (Figure 2B,C). However, the number of Annexin V-positive cells was significantly higher in CSS spheres (19.32 ± 0.31) as compared to PC3M spheres (2.45 ± 2.37) after 24 h incubation with anti-CD19 CAR-T cells (*p* < 0.001) (Figure 2C,D).

### 3.2. CSS-Conditioned Media Displays a Distinct Cytokine Profile

To investigate the initial immune profiles of all cell types in the study, we evaluated the cytokine levels in the conditioned media of cancer and stroma spheres, as well as the culture of anti-CD19 CAR-T cells. A total of 38 cytokines were analyzed and categorized in the conditioned media.

**Figure 3:**
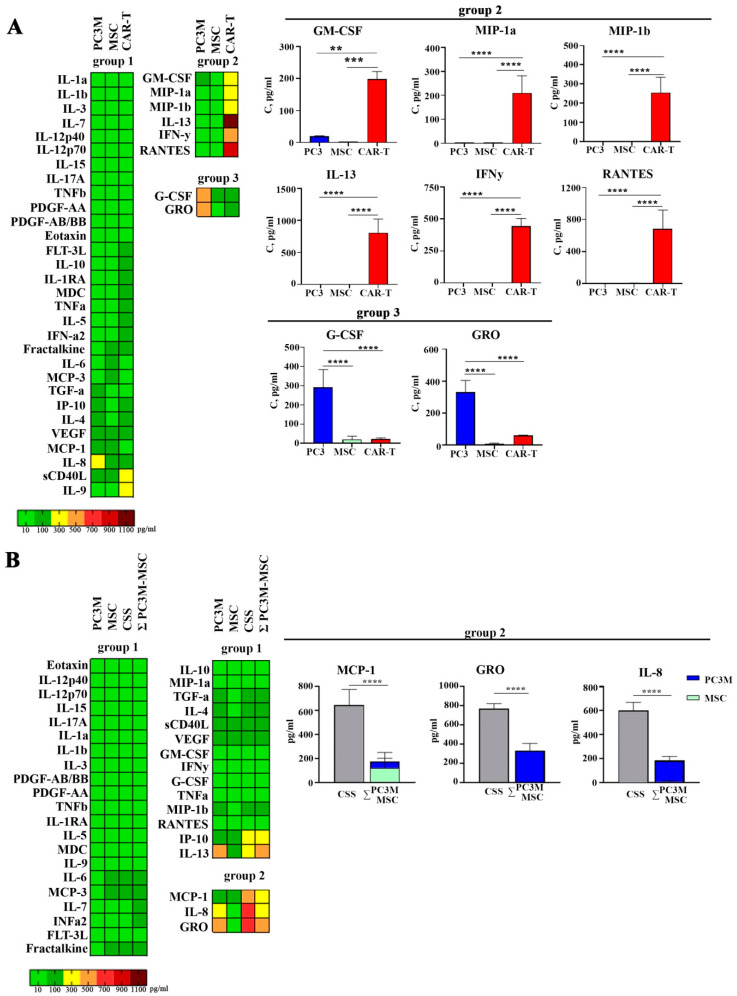
Analysis of cytokine and chemokine concentrations in the conditioned media: (**A**) analysis in the conditioned media of PC3M and MSC spheres, anti-CD19 CAR-T cell culture; (**B**) analysis in the conditioned media of PC3M and MSC spheres, and complex CSSs (*p*-values are indicated as **—≤0.01, ***—≤0.005, ****—≤0.0001).

First, we ranked the cytokines according to the alterations observed in the different cell types used in the study (PC3M, MSC, CAR-T) (Figure 3A). The levels of cytokines IL1a, IL1b, IL3, IL-7, IL-12p40, IL-12p70, IL-15, IL-17A, TNFb, PDGF-AA, PDGF-AA/AB, Eotaxin, FLT-3L, IL-10, IL-1Ra, MDC, TNFa, IL-5, INFa2, IL-9, Fractalkine, IL-6, MCP-3, TGFa, IP-10, IL-4, VEGF, MCP-1, IL-8, sCD40L and IL-9 were low and did not differ significantly in conditioned media of PC3M and MSC spheres or anti-CD19 CAR-T cells (Figure 3A, group 1).

In contrast, GM-CSF, MIP1a, MIP1b IL-13, IFNγ and RANTES were secreted by anti-CD19 CAR-T cells at significantly higher levels than by other cell types in the study (Figure 3A, group 2).

G-CSF and GRO were detected in PC3M-conditioned media at significantly higher concentrations as compared to MSCs and anti-CD19 CAR-T conditioned media (Figure 3A, group 3).

Next, we investigated the alterations in cytokine profiles induced by the co-culture of MSC and PC3M cells in a CSS. The arithmetic sum (Ʃ) of the cytokine amounts in the conditioned media of spheres formed by MSCs and PC3M was compared with the cytokine levels detected in the CSS (Figure 3B).

Significantly higher levels of MCP-1, GRO, and IL-8 were detected in CSS culture, as compared to the Ʃ of two single-cell type sphere cultures: (Figure 3B, group 2).

### 3.3. Spheres Show Distinct Immunological Responses to Anti-CD19 CAR-T Cell Treatment

**Figure 4:**
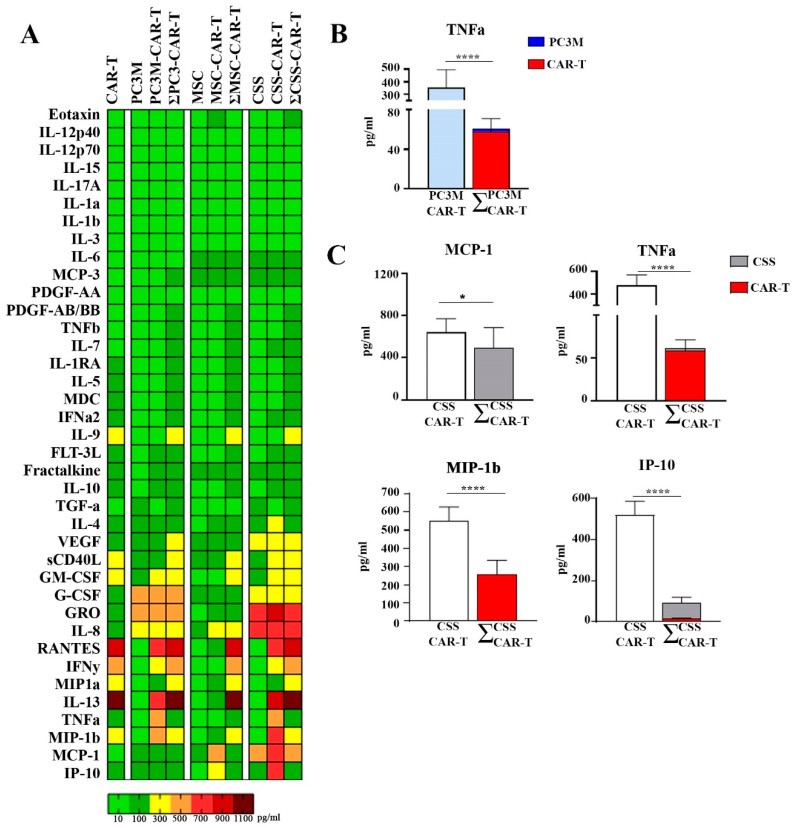
Analysis of cytokine and chemokine concentrations in the conditioned media of spheres treated with anti-CD19 CAR-T cells for 6 h: (**A**) heatmap of cytokine and chemokine levels analyzed via multiplex immunoassay; (**B**) the TNFa level in the conditioned media of anti-CD19 CAR-T cell-treated PC3M spheres, and the arithmetic sum (Ʃ) of the cytokine amounts; (**C**) the MCP-1, TNFa, MIP-1b and IP-10 levels in the conditioned media of anti-CD19 CAR-T cell-treated CSSs and the arithmetic sum (Ʃ) of the cytokine amounts. *—≤0.05, ****—≤0.0001.

To investigate the initial immune response of tumor and stromal cells upon co-cultivation with anti-CD19 CAR-T cells, we evaluated the cytokine levels in the conditioned media of spheres after 6 h of incubation with anti-CD19 CAR-T cells. The treatment of spheres with CAR-T cells resulted in the hyperproduction of various immunological factors in a cell-dependent manner (Figure 4A).

Incubation of PC3M spheres with anti-CD19 CAR-T cells led to an elevation of TNF-α compared to the expected yield based on the sum of anti-CD19 CAR-T-treated MSC and PC3M sphere cultures (Figure 4B). CSS culture with anti-CD19 CAR-T resulted in increases in TNF-α, MIP-1β, MCP-1, and IP-10 (Figure 4C).

**Figure 5:**
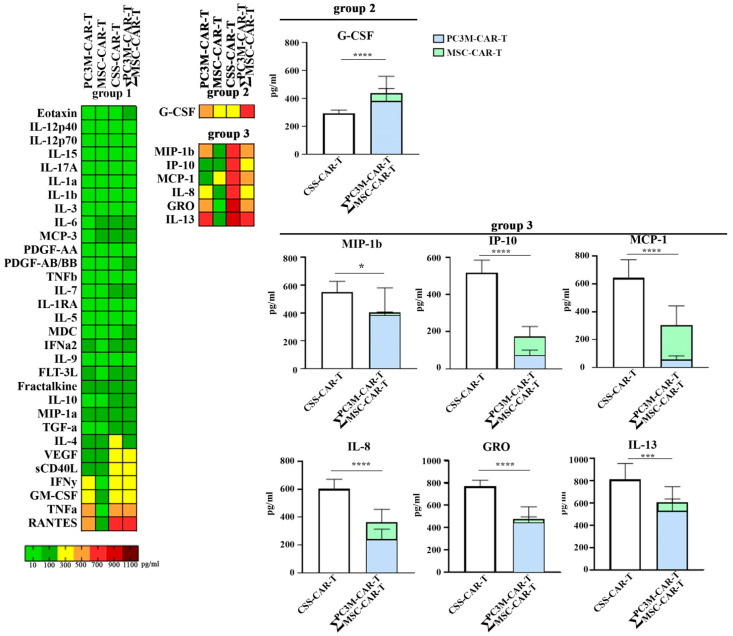
Immunological response of PC3M, MSC spheres, and CSSs to CAR-T treatment. (*p*-values are indicated as *—≤0.05, ***—≤0.005, ****—≤0.0001).

Furthermore, the arithmetic sum (Ʃ) of cytokine levels detected in the conditioned media of anti-CD19 CAR-T cell-treated MSC and PC3M spheres was compared with the cytokine level detected in the CSS (Figure 5). The arithmetic sum was higher than the values detected in CSS for G-CSF, which may indicate the utilization of the protein by one of the two cell types (Figure 5, group 2).

Notably, levels of MIP1b, IP10, MCP1, IL-8, GRO, and IL13, exceeded expected values and were significantly higher in anti-CD19 CAR-T treated CSS conditioned media than Ʃ of secreted cytokines from PC3M and MSC conditioned media (Figure 5, group 3).

## 4. Discussion

The use of CART therapy is currently being actively studied not only for hematological malignancies but also for solid tumors. The use of tumor spheres allows us to create a test system that will also be relevant for solid cancer therapy studies. Several previous studies have utilized tumor sphere and organoid cultures for CAR-T treatment. For example, anti-HER2 CAR-T cells mediated lysis of pancreatic ductal adenocarcinoma (PDAC) spheres in 215 out of the 354 cultures [38]. The lysis was associated with a significant increase in IFNγ levels. Notably, an increase in stem cell proportion was demonstrated in PDAC spheres upon CAR-T cell exposure.

In our study, we employed a co-culture of prostate and stroma cells in a sphere format to obtain CSS. The rationale was twofold: to analyze the contribution of mesenchymal cells to CART-associated CRS and ICANS, and to assess the applicability of the CSS model for CART drug toxicity studies. We analyzed levels of 41 cytokines; however, three cytokines (IL-2, FGF2, EGF) were excluded from the analysis due to the presence of their corresponding recombinant proteins in the culture media.

### 4.1. CSS Immune Profile Display Tumor Promoting and Immunosuppressive Phenotype

Initially, we demonstrated that CAR-T cells induce apoptosis more efficiently in CSS than in PC3M spheres alone (Figure 2C,D).

Multiplex analysis of conditioned media from single cell type cultures revealed high levels of G-CSF (granulocyte colony-stimulating factor) and GRO (growth-regulated alpha protein;C-X-C motif chemokine 1) secretion by PC3M spheres (Figure 3A, group 3). The role of GRO expression in prostate cancer cells was revealed in the murine TRAMP-C2 cell model, lacking expression of CXCL8 and overexpressing CXCL1 [39]. GRO overexpression cells showed elevated invasion and migration ability, indicating the possible activation of these characteristics in PC3M cells growing under conditions of large (>100 µm) dense spheres. High levels of G-CSF also support cancer cell metastasis, proliferation, and the maintenance of cancer stem cells [40].

CAR-T cell conditioned media contained high levels of GM-CSF, IFNγ, IL-13, MIP-1α (macrophage inflammatory protein 1-alpha, CCL3), MIP-1β (macrophage inflammatory protein 1-beta, CCL4), and RANTES (regulated on activation, normal T-cell expressed and secreted, CCL5) (Figure 3A, group 2). Activated T-cells release GM-CSF and IFNγ. IFN-γ enhances the ability of macrophages to present antigens, secrete inflammatory cytokines, and produce high levels of oxygen and nitrogen intermediates [41]. High levels of single GM-CSF have been associated with a high risk of CRS, and blocking GM-CSF in humanized mouse models resulted in reduced rates of CRS and ICANS [42]. IL-13 has been shown to suppress apoptosis in activated CD4+ cells [43]. Remarkably, Il13 is also a cancer immunosurveillance factor then presented in tumor microenvironment [44]. MIP-1α, MIP-1β, and RANTES are beta chemokines secreted by CD8+ cells. In virus-infected cells beta chemokines induce non-cytolytic suppression of viral replication in CD4+ cells [45]. We can hypothesize that the production of beta chemokines is a response from non-transduced CD8+ T cells to the presence of CD4+ T cells transduced with CAR-carrying lentivirus; however, this theory requires experimental validation. In cancer studies, beta chemokines have been shown to play a protumorogenic role by recruiting CCR2+ monocytes to the tumor site, which subsequently differentiate into tumor-associated macrophages (TAMs) [46]. Furthermore, Dorner and colleagues performed a single-cell study and showed that MIP-1α, MIP-1β, and RANTES are co-secreted with IFNγ in activated T-cells [47]. The authors claim that three chemokines together serve as coactivators of macrophages and can affect the activity of NK cells.

Our next step was to obtain CSS and analyze the immunological profile of the conditioned media resulting from their cultivation. The immunological profile of CSS, composed of tumor and stromal cells, was characterized by a significant increase in the secretion of IL-8, GRO, and MCP1(Figure 3B, group 2). It has been shown that IL-8 can be produced by both tumor and stromal cells. MSCs are known to support tumor cell growth and survival by secretion of regulatory paracrine factors. IL-8 has been shown to be an MSC-derived angiogenesis promotion factor in colorectal cancer studies [48]. At the same time, tumor cells produce IL-8 to recruit myeloid cells to form tumor-promoting microenvironment [49].

All three subtypes of growth-regulated oncogene (GRO)—GRO-α/CXCL1, GRO-β/CXCL2, and GRO-γ/CXCL3—bind to the CXCR2 receptor. The level of GRO-α is elevated in various cancers and is associated with unfavorable patient prognosis in hepatocellular and pancreatic cancers [50,51]. Overexpression of GRO-α in hepatoma cell lines has been shown to enhance cell proliferation and invasion [51].

MCP-1 is a crucial immunological regulator that controls the migration, activation, and polarization/differentiation of immune cells. Both stromal and cancer cells produce MCP-1. In osteosarcoma, breast, ovarian, and prostate, this chemokine has been shown to induce invasion and migration of cancer cells (reviewed in [52]). In mice models of contact hypersensitivity, MCP-1 pre-treated MSCs demonstrated the ability to suppress T cell proliferation, reduce the secretion of pro-inflammatory cytokines, and polarize macrophages towards the M2 phenotype [53].

Taken together, all presented evidence suggests that the co-culture of tumor and mesenchymal cells in a sphere format creates a microenvironment that exerts an immunosuppressive effect, ultimately enhancing the survival and metastatic potential of tumor cells.

### 4.2. CAR-T Cell Treatment of CSS Leads to Secretion of CRS and ICANS-Associated Factors

Treatment of PC3M spheres with CAR-T cells led to an elevation of tumor necrosis factor alpha (TNFα) (Figure 4B). TNFα is produced by T cells upon binding to CD19 on the surface of PC3M cells, indicating the activation of the T-cell response. At the same time, TNFα has been recognized as a main cytokine involved in endothelial cell activation, and its hyperproduction has been associated with the progression of CRS [54]. This finding was further confirmed by TNFα blocking experiments using adalimumab, which resulted in decreased activation of human umbilical vein endothelial cells (HUVECs) [54].

Co-culture of CSS with CAR-T cells resulted in increased levels of TNFa, MCP-1, MIP-1β, and IP-10 (Figure 4C). Among these cytokines, MCP-1 demonstrated superior predictive value for severe CRS, with a sensitivity of 100% and specificity of 95%, across different grades of CRS in an anti-CD19 CAR-T study (levels of IFNγ, IL-6, IL-8, IL-10, IL-15, MCP-1, TNF receptor p55 (TNFRp55), and MIP-1β have been evaluated) [12].

IP-10 acts as a chemoattractant for immune cells [55] and also stimulates the proliferation and antitumor activity of CAR-T cells [56]. Serum levels of IP-10 show a positive correlation with progression-free survival in multiple myeloma patients. At the same time, IP-10 has been identified as a marker for ICANS and has been detected in the cerebrospinal fluid of CAR-T-treated patients [3].

The co-culture of spheres with CAR-T cells revealed that the level of G-CSF was significantly lower in CSS culture than expected based on the sum of MSC and PC3M cultures (Figure 5, group 2). At the same time, multiplex analysis data indicate that most of the G-CSF is produced by prostate adenocarcinoma cells (Figure 3A, group 3). Previous studies have shown that G-CSF can promote MSC proliferation under both in vitro and in vivo conditions [57]. Thus, we can conclude that G-CSF is secreted by PC3M cells and plays a critical role in the maintenance of MSC proliferation in complex CSS.

When comparing the cytokine profiles of all CAR-T treated spheres, we identified factors that were significantly increased only when heterogeneous CSS was used. CSS treatment with CAR-T resulted in significant elevation of IL-8, MCP-1, IP10, MIP-1β, GRO, and IL13 (Figure 5, group 3). Among listed cytokines IL-8, MCP-1, and IP-10 have been associated with CRS, ICANS, or both.

## 5. Conclusions

When T-cells are incubated with cancer cells, we observe T-cell-intrinsic cytokine secretion, which to a certain extent, may predict in vivo immune toxicity by level of hyperproduction of TNFα, IFNγ, MCP-1, and others. Secondary inflammatory mediators are produced by the patient’s endogenous cells and play a more crucial role in the development of side effects; for example, data on IL-6 production by monocytes is well documented. The role of MSCs in CAR-T-associated immune and neurological toxicity remains to be fully elucidated.

In our study, we demonstrate that the cancer-stromal cell crosstalk modulates the immune response to CAR-T therapy, highlighting a synergistic effect and cooperation among the cells within a CSS. The presented data indicate that complex cancer-stroma spheres represent a powerful tool to study CAR-T-associated toxicity, as they show the production of CRS and ICANS-associated cytokines, which were not observed in single cell-type spheres, neither in cancer nor stromal cells.

It is important to note that a two-cell system cannot fully replicate the complexity of a living organism. In our study, we do not observe inflammatory cytokines induced by monocytes, endothelial cells, or other participants in CRS. Nevertheless, our study emphasizes the importance of considering the role of MSCs in the development of the immune response and demonstrates the utility of a new CSS in vitro model for investigating the toxicity of novel CAR-T therapeutics.

## Data Availability

Data are contained within the article.
